# Nasopharyngeal carcinoma: United Kingdom National Multidisciplinary Guidelines

**DOI:** 10.1017/S0022215116000517

**Published:** 2016-05

**Authors:** R Simo, M Robinson, M Lei, A Sibtain, S Hickey

**Affiliations:** 1 Department of Otolaryngology – Head and Neck Surgery, Guy's and St Thomas’ Hospital NHS Foundation Trust, Guy's, King's and St Thomas’ Medical and Dental School, London, UK; 2 School of Dental Sciences, Newcastle University, Newcastle-upon-Tyne, UK; 3 Department of Oncology, Guy's and St Thomas’ Hospital NHS Foundation Trust, London, UK; 4 Department of Clinical Oncology, St Bartholomew's Hospital, London, UK; 5 Department of Otolaryngology-Head and Neck Surgery, Torbay Hospital, Torquay, UK

## Abstract

This is the official guideline endorsed by the specialty associations involved in the care of head and neck cancer patients in the UK. Although much commoner in the eastern hemisphere, with an age-standardised incidence rate of 0.39 per 100 000 population, cancers of the nasopharynx form one of the rarer subsites in the head and neck.^1^ This paper provides recommendations on the work up and management of nasopharyngeal cancer based on the existing evidence base for this condition.

**Recommendations:**

• Patients with nasopharyngeal carcinoma (NPC) should be assessed with rigid and fibre-optic nasendoscopy. (R)

• Nasopharyngeal biopsies should be preferably carried out endoscopically. (R)

• Multislice computed tomographic (CT) scan of head, neck and chest should be carried out in all patients and magnetic resonance imaging (MRI) where appropriate to optimise staging. (R)

• Radiotherapy (RT) is the mainstay for the radical treatment for NPC. (R)

• Concurrent chemoradiotherapy offers significant improvement in overall survival in stage III and IV diseases. (R)

• Surgery should only be used to obtain tissue for diagnosis and to deal with otitis media with effusion. (R)

• Radiation therapy is the treatment of choice for stage I and II disease. (R)

• Intensity modulated radiation therapy techniques should be employed. (R)

• Concurrent chemotherapy with radiation therapy is the treatment of choice for stage III and IV disease. (R)

• Patients with NPC should be followed-up and assessed with rigid and/or fibre-optic nasendoscopy. (G)

• Positron emission tomography–computed tomography (PET–CT), CT or MRI scan should be carried out at three months from completion of treatment to assess response. (R)

• Multislice CT scan of head, neck and chest should be carried out in all patients and MRI scan whenever possible and specially in advanced cases with suspected recurrence. (R)

• Surgery in form of nasopharyngectomy should be considered as a first line treatment of residual or recurrent disease at the primary site. (R)

• Neck dissection remains the treatment of choice for residual or metastatic neck disease whenever possible. (R)

• Re-irradiation should be considered as a second line of treatment in recurrent disease. (R)

## Introduction

Nasopharyngeal carcinoma (NPC) is a squamous cell carcinoma (SCC) arising from the mucosal surface of the nasopharynx. The most common site is the fossa of Rosenmüller which is a recess just medial to the medial crura of the eustachian tube. Nasopharyngeal carcinoma is frequent in patients of Southern Chinese, Northern African and Alaskan origin. The incidence in the Hong Kong population is between 20 and 30 per 100 000 inhabitants a year, but in Western countries the adjusted incidence is very low; around 1 per 100 000 per annum.^2^

## Aetiology and risk factors

The Epstein–Barr virus (EBV) and consumption of salted fish containing *dimethylnitrosamine* have been implicated in its aetiology. Genetic alterations include deletion of chromosomal regions at 1p, 14q, 16p and amplification of 4q and 12q.

## Clinical presentation

Nasopharyngeal carcinoma is more common in men than in women (3:1), with a median age at presentation of 50 years. The most common symptoms are:

• Nasal obstruction
• Epistaxis
• Conductive hearing loss secondary to otitis media with effusion (OME) due to eustachian tube orifice obstruction
• Cranial nerve neuropathies secondary to skull base invasion (cranial nerves III, IV, V and VI)
• Neck lumps and swellings due to cervical lymph node metastasis, which is usually in the upper levels of the neck and often bilateral due to the midline lymphatic drainage of the tumour.

## Assessment and staging

### Clinical assessment

Full history and otorhinolaryngological examination with rigid or fibre-optic nasendoscopy in the out-patient setting should be performed. Examination under anaesthetic with endoscopic assessment and biopsy of the nasopharyngeal abnormality is mandatory with targeted biopsies of the fossa of Rosenmüller, when indicated. Biopsies should be preferably done after staging scans to avoid false artefacts.

### Pathologic considerations

Histological examination is required for the definitive diagnosis. Fine needle aspiration cytology (FNAC) can be used as an adjunct for staging neck disease and distant metastases.

Nasopharyngeal carcinoma (NPC) comprises three histological types: non-keratinising carcinoma (incorporating differentiated and undifferentiated subtypes), keratinising carcinoma and basaloid SCC. All NPCs share morphological and immunohistochemical features of squamous differentiation to varying degrees.

Non-keratinising carcinoma is by far the most common type in both high and low incidence areas. The diagnosis of keratinising carcinoma and basaloid SCC is facilitated by the identification of malignant epithelium that shows overt keratinisation. By contrast, non-keratinising carcinoma has subtle morphological features that are often obscured by a dense lymphoid stroma, from which the synonym lymphoepithelial carcinoma is derived. Immunohistochemistry is required to identify the production of keratin intermediate filaments. Antibodies AE1/AE3 and MNF116 can be used to detect of a broad range of keratin molecules and when the malignant cells are positive they support a diagnosis of carcinoma. Cytoplasmic expression of cytokeratins 5/6 and nuclear expression of p63 can be used as evidence of squamous differentiation. Epstein–Barr virus (EBV) has been recognised as a primary aetiological agent in non-keratinising NPC. The presence of EBV is most reliably detected using in situ hybridisation for EBV encoded early RNA (EBER), whereas the expression of latent membrane protein-1 is less sensitive and is positive in about a third of cases.

Serological markers of EBV infection are detected in almost all cases of non-keratinising carcinoma, but have limited diagnostic utility. They can be used as an adjunct to monitor disease progression and response to treatment, although the practical clinical use remains unproven. Detection of immunoglobulins to viral capsid antigen and early antigens are the most commonly used tests. In addition, the detection of EBV nucleic acid (DNA and RNA) in serum and plasma, using quantitative polymerase chain reaction techniques, has been developed to aid disease surveillance.

Human papilloma virus (HPV) has been recognised as a primary aetiological agent in a subset of head and neck SCCs, primarily oropharyngeal in origin. A number of studies have reported HPV-positivity in NPCs, either with or without concurrent EBV association. The clinical significance of this relationship has not yet been established.

### Imaging considerations

Staging investigations should include multislice computed tomography (CT) scan of the head, neck and chest. Magnetic resonance imaging (MRI) scans of the skull base are useful especially in locally advanced tumours. The use of positron emission tomography–computed tomography (PET–CT) should be reserved for patients with a suspected occult primary tumour in the nasopharynx and should be carried out before diagnostic procedure. Ultrasound guided FNAC of suspected cervical lymph node metastases is recommended, if they cannot be definitively labelled as malignant on cross-sectional imaging.

### Staging

See Tables I–IV.

**Table I:** Primary tumour (T)

T1	Tumour confined to nasopharynx or extends to oropharynx and/or nasal cavity
T2	Tumour with parapharyngeal extension
T3	Tumour invades bony structures and/or paranasal sinuses
T4	Tumour with intracranial extension and/or involvement of cranial nerves, infratemporal fossa, hypopharynx, orbit or masticator space

**Table II:** Regional lymph node metastases (N)

Nx	Regional lymph nodes cannot be assessed
N0	No regional lymph node metastasis
N1	Unilateral metastasis in lymph nodes, 6 cm or less in the greatest dimension, above the supraclavicular fossa
N2	Bilateral metastasis in lymph nodes, 6 cm or less in the greatest dimension, above the supraclavicular fossa.
N3	Metastasis in a lymph node greater than 6 cm in dimension or extension to the supraclavicular fossa
N3a	Greater than 6 cm in dimension
N3b	Extension to the supraclavicular fossa

**Table III:** Distant metastasis (M)

Mx	Distant metastasis cannot be assessed
M0	No distant metastasis
M1	Distant metastasis

**Table IV:** Stage grouping

Stage 0	Tis	N0	M0
Stage I	T1	N0	M0
Stage II	T2	N0	M0
	T1	N1	M0
	T2	N1	M0
Stage III	T1	N2	M0
	T2	N2	M0
	T3	N0	M0
	T3	N1	M0
	T3	N2	M0
Stage IVa	T4	N0	M0
	T4	N1	M0
	T4	N2	M0
Stage IVb	Any T	N3	M0
Stage IVc	Any T	Any N	M1

**Recommendations**

• Patients with NPC should be assessed with rigid and fibre-optic nasendoscopy (R)
• Nasopharyngeal biopsies should preferably be carried out endoscopically (R)
• Multislice CT scan of head, neck and chest should be carried out in all patients and MRI where appropriate to optimise staging (R)

## Management

### Radiotherapy

Radiotherapy (RT) is the mainstay for the radical treatment for nasopharyngeal carcinoma (NPC).^3^ The anatomical location, propensity for loco-regional spread, and proximity of critical structures makes wide field surgical treatment unacceptably morbid as a first line option. The therapeutic ratio of RT is improved by the addition of synchronous chemotherapy (CT) and advances in radiation delivery techniques, both of which may help to achieve improved disease control and survival along with lower rates of long-term toxicity. Intensity modulated radiotherapy (IMRT) delivery techniques allow concavities to be created in the RT dose distribution, which is particularly useful for the treatment of head and neck cancer. It facilitates improved dosimetric coverage of the primary tumour volume, particularly in the pharyngeal recesses, and sparing of normal organs, including the parotid gland, substantially reducing long-term xerostomia, thereby improving quality of life.

Proton beam therapy is a newly emerging technology which may further improve radiation dose conformality. Evidence to support a difference in outcomes compared to those achieved with conventional radiotherapy is lacking. Currently, nasopharyngeal tumours in paediatric, teenage and young adult patients, are included as permitted indications in the NHS England Proton Programme and these patients should be referred accordingly.

Radiotherapy is also useful in the palliative setting. It can be used to treat symptomatic metastases and local disease in the presence of widespread metastases when aggressive local therapy is clinically inappropriate.

**Recommendation**

• Radiotherapy is the mainstay for the radical treatment for NPC (R)

### Chemotherapy

There is evidence confirming significant improvement in overall survival (OS) in the patients treated concurrently with chemoradiotherapy for NPC as compared to RT alone. The addition of chemotherapy has been shown to confer a small, but significant benefit in OS and event-free survival (EFS). A meta-analysis of eight randomised trials and 1753 patients reported an 18 per cent reduction in the hazard ratio for death with the use of any form of chemotherapy with RT, corresponding with an absolute survival benefit of 6 per cent at five years. The greatest benefit was observed with concomitant chemotherapy. The roles of neo-adjuvant and adjuvant chemotherapy are more controversial with no proven survival advantage but confirmed EFS benefit with neo-adjuvant chemotherapy. Adjuvant chemotherapy after RT is less well tolerated and benefits are still unproven. Cisplatin-based chemotherapy is used concurrently with radiation and combination of cisplatin and fluorouracil may be used in the neo-adjuvant setting, in selected cases. Platinum-based chemotherapy has been effective in palliation of recurrent and metastatic NPC. Single centre (level 2) studies have reported activity with the use of capecitabine, gemcitabine and taxanes as single agent or in combination with platinum for second- and third-line treatments for metastatic disease.

A randomised trial including 803 patients with stages III–IVB NPC compared induction and concurrent chemotherapy, replacement of fluorouracil with oral capecitabine and/or accelerated RT found no benefit by changing to an induction–concurrent sequence.^4^^–^^7^

**Recommendation**

• Concurrent chemoradiotherapy offers significant improvement in OS in stage III and IV diseases (R)

### Primary surgery

Surgery is only used in the following scenarios:

• To obtain tissue for diagnosis. Contact endoscopic diagnosis of NPC remains experimental
• To obtain tissue from clinically involved neck nodes using FNAC or core biopsy. If these techniques are non-diagnostic, open biopsy can be used. In cases with obvious fungation open biopsy is the method of choice
• To deal with OME.

**Recommendation**

• Surgery should only be used to obtain tissue for diagnosis and to deal with otitis media with effusion (R)

## Treatment recommendations

### Stages I and II

Patients with early disease can be treated with RT alone, resulting in disease free survival rates of 90 and 84 per cent. The dose to the primary tumour should be equivalent to 70 Gy in 2 Gy fractions and at least 50 Gy in 2 Gy fractions to the bilateral neck and other sites of potential microscopic spread. Intensity modulated radiotherapy techniques should be used. Evidence of benefit from the addition of chemotherapy to RT in early disease is lacking.

Intermediate stage II disease can be treated with RT alone (T2N0M0), but most cases are treated with combination chemoradiotherapy. Intensity modulated radiotherapy should be considered mandatory. A dose of 70 Gy is recommended to the primary, 66–70 Gy to gross disease in lymph nodes and 50 Gy to the bilateral neck and other sites of potential microscopic spread. Radiobiological equivalents are given if a fraction size other than 2 Gy is employed, for example with intensity modulation. The most commonly used chemotherapy schedule is cisplatin 100 mg/m^2^ on days 1, 22 and 43 of RT based on the United States Intergroup Study 0099. Weekly cisplatin at a dose of 40 mg/m^2^ is effective, but has not been compared with the standard regimen in a randomised study. It can be considered for older patients and/or those with significant comorbidities.

### Stages III and IV

Concurrent chemoradiotherapy is the standard of care for advanced nasopharyngeal cancers. This improves OS by up to 6 per cent at five years compared with radical RT. A dose of 70 Gy (2 Gy per fraction) with concurrent cisplatin chemotherapy is recommended. Several trials have explored the role of neo-adjuvant chemotherapy, with a recent meta-analysis confirming an improvement in disease-free survival, whilst having no effect on OS. Radiotherapy target volume definition must include gross tumour (clinical, endoscopic and radiological), the nasopharynx and the pterygopalatine fossa, the base of skull and clivus, the posterior part of sphenoid sinus, the posterior third of the nasal cavity and the maxillary sinus, retropharyngeal lymph nodes and parapharyngeal space. Prophylactic irradiation must include uninvolved level I–V nodal areas.

IMRT is used with either fixed gantry linear accelerator-based techniques (fixed field or volumetric arc) or with helical tomotherapy techniques with confirmed benefits in preserving parotid gland function. Studies are currently exploring the role of further dose escalation with IMRT to improve local control.^8^^–^^10^

Surgical treatment is reserved for salvage following chemoradiotherapy failure.

**Recommendations**

• Radiation therapy is the treatment of choice for stage I and II diseases (R)
• Intensity modulated radiation therapy techniques should be employed (R)
• Concurrent chemotherapy with radiation therapy is the treatment of choice for stage III and IV diseases (R)

## Assessment of treatment response and follow-up

Assessment of treatment response and follow-up is imperative in nasopharyngeal carcinoma (NPC). Patients should be assessed clinically with endoscopic examination and neck palpation. Currently there is no consensus on the best mode of radiological assessment to determine completeness of response to treatment. PET–CT, CT or MRI follow-up scans have been adopted in some centres at three months and at a year from completion of treatment.

Following treatment, it can take up to three months for NPC to disappear histologically. Post-treatment disease can be monitored using biopsies. However, accurate interpretation of the material can be confounded by persistent areas of degenerate tumour, the biological significance of which needs to be assessed in the context of the temporal relationship to treatment. Furthermore, tissue changes in the radiation field can also mimic residual disease and need to be interpreted with caution. The presence of morphologically viable malignant cells with evidence of EBER by in situ hybridisation is strongly suggestive of residual disease. If a biopsy contains carcinoma, repeat sampling two weeks apart is recommended and remission is defined as two sequential negative biopsies. The recommended follow-up strategy is addressed elsewhere in the guidelines.

**Recommendations**

• Patients with NPC should be followed-up and assessed with rigid and/or fibre-optic nasendoscopy (G)
• Positron emission tomography–computed tomography, CT or MRI scan should be carried out at three months from completion of treatment to assess response (R)
• Multislice CT scan of head, neck and chest should be carried out in all patients and MRI scan whenever possible and specially in advanced cases with suspected recurrence (R)

## Management of residual and recurrent disease

### Surgery

Chemoradiotherapy or RT resistant tumours may be amenable to salvage surgery to the primary site or the neck. Surgery for recurrence is associated with less morbidity than re-irradiation of recurrent disease. Nasopharyngectomy and/or neck dissection should be the first option for locoregional residual and recurrent disease. When surgery is not possible either palliative chemotherapy or re-irradiation should be considered.

#### Surgery to the primary site

Endoscopically guided microwave coagulation of small volume (rT1) recurrent disease has been described as having low morbidity with OS and local progression-free survival of 93.6 and 90.7 per cent at five years.

The likelihood of successful surgical excision diminishes in proportion to the size and extent of the recurrent and/or persistent disease at the primary site. Transcranial approaches are associated with high morbidity. Transnasal and transantral approaches provide poor access to the paranasopharyngeal space. Experience with combined transoral and transnasal endoscopic resection is increasing and may become the favoured approach for small lesions, because of the low associated morbidity. Robotic surgical techniques, although used in only a few studies, have been shown to offer equivalent local control in highly selected cases.

The anterolateral approach with maxillary swing (facial translocation) allows access to the nasopharynx and paranasopharyngeal space and has a local control rate of up to 62 per cent. Palatal fistulae occur in 20–25 per cent of patients, whilst 60 per cent have some degree of trismus. A lateral approach with radical mastoidectomy and exposure of the infratemporal fossa after mobilisation of the internal carotid, trigeminal nerve and floor of the middle cranial fossa has been described. Its use is associated with considerable risk of morbidity.^11^^–^^13^

#### Surgery to the neck

Re-irradiation of the involved neck to treat persistent and/or recurrent disease carries a high risk of tissue necrosis and fibrosis. Persistent or recurrent nodal disease following chemoradiotherapy demonstrates a high incidence of extracapsular extension (54–65 per cent). For this reason salvage radical neck dissection (with the placement of brachytherapy tubes for after loading where there is extensive disease), remains the treatment of choice. It may be necessary to excise involved skin and repair with pedicled or microvascular flaps. Transferred tissue flaps should be placed so as to overlie brachytherapy tubes as they are often more tolerant of irradiation than previously irradiated skin. Salvage neck dissection gives up to 66 per cent five-year local control of disease.^14^

### Non-surgical options

#### Re-irradiation

Local nasopharyngeal recurrences respond better to re-irradiation than other sites.^15^ The scope for re-irradiation depends on the tumour volume, current T stage and the disease free interval or time since primary irradiation. The dose that can be delivered depends on the dose received by adjacent critical organs, time since initial irradiation and the technique of RT delivery. In general, a dose of greater than 50 Gy needs to be deliverable for re-irradiation to be worthwhile. This is more achievable using highly conformal techniques, including IMRT, intracavitary and interstitial brachytherapy, stereotactic radiosurgery, fractionated stereotactic RT or proton beam therapy. Overall survival rates of 60 per cent at five years have been reported. The toxicity rate for re-irradiation is significant. A prognostic scoring system for locally recurrent nasopharyngeal carcinoma (NPC) has been validated. When re-irradiation is not possible, then palliative chemotherapy should be considered.

#### Conventional chemotherapy

Palliative systemic chemotherapy is the central component of the treatment for metastatic disease.^16^ Cisplatin-based chemotherapy produces response rates of up to 80 per cent in chemotherapy-naive patients resulting in median survival rates of up to 15 months. There are no randomised comparisons of different chemotherapy schedules. Whilst cisplatin and fluorouracil remain the most widely used combinations, the gemcitabine and cisplatin doublet has also been shown to produce high response rates and be well tolerated, and can be considered above other agents that have higher toxicity. Triplet combinations also produce higher response rates, but at the cost of higher toxicity. The decision to give palliative chemotherapy should take into account previous therapy and the performance status of the patient.

### Molecular therapies and immunotherapy

There is no evidence for the routine use of molecular therapies for metastatic NPC outside clinical trial settings. Phase II trials have demonstrated limited activity in the second- and third-line settings. The utility of immunotherapy based either adoptive or active means against Epstein–Barr virus (EBV) antigens, remains investigational.

**Recommendations**

• Surgery in form of nasopharyngectomy should be considered as a first-line treatment of residual or recurrent disease at the primary site (R)
• Neck dissection remains the treatment of choice for residual or metastatic neck disease whenever possible (R)
• Re-irradiation should be considered as a second line of treatment in recurrent disease (R)

## Treatment outcomes

### Stages I and II

Five-year OS rates of 90 per cent for stage I and 84 per cent for stage II have been reported from a review of 2687 patients from Hong Kong, based on the AJCC 1997 staging. Data for non-endemic regions are sparse given the relative rarity of the condition in these areas. Serum Epstein–Barr virus (EBV) DNA copies before treatment have been shown to have prognostic significance; for stages I and II <4000 copies per ml have a 91 per cent survival at five years and >4000 copies per ml have a 64 per cent five-year survival.

### Stages III and IV

Chemoradiotherapy regimes have improved the OS of nasopharyngeal carcinoma (NPC) patients from 77 to 81 and  56 to 62 per cent at two and five years, respectively. The benefit for chemotherapy was not lost for advanced stage disease.

Recent studies using simultaneous integrated boost delivered following neo-adjuvant chemotherapy with IMRT, in locally advanced NPC, have suggested local progression free and distant metastases free survival rates of 80–90 per cent. Accelerated RT schedules or post-RT brachytherapy boost have produced excellent local control rates, but more studies and longer follow-up data are awaited to confirm the benefits.

Patients with systemic metastases have been treated with cisplatin containing regimes with response rates ranging from 40 to 80 per cent, and median survival of about 14 months.

## Controversies

### The role of ventilation tubes in the management of the middle-ear effusion in nasopharyngeal cancer patients

The rate of complications (otalgia and otorrhoea) is higher if grommets are inserted after RT. Eustachian tube function may improve in an irradiated patient up to five years after RT. However, if an effusion is present or develops during RT tubal function remains poor. Grommets bypass tubal obstruction, but may exacerbate the inflammatory process. Up to 29 per cent of patients will develop non-healing perforation of the tympanic membrane, if grommets are inserted during or after RT and 49 per cent will go onto develop intermittent otorrhoea. Middle ear effusion arising during or after RT is best managed using repeated paracentesis, aspiration and a hearing aid. Grommets should be used as a last resort.^17^^,^^18^

### Salvage surgery for local recurrence

The maxillary swing nasopharyngectomy approach has now been adopted as an adequate mode of salvaging patients with recurrent nasopharyngeal carcinoma (NPC) with survival rates of up to 73 per cent in selected cases. The use of a purely endoscopic approach has been attempted, without evidence of any benefit. The controversy arises mainly on the accurate assessment, patient selection and extent of the resection, weighing the benefits of the procedure against their morbidity.

### Salvage treatments for recurrent disseminated disease

Isolated, potentially surgically treatable metastases in NPC are rare and only limited reported cases specific for NPC are available in the literature.

### The role of neo-adjuvant and adjuvant chemotherapy

Recent meta-analyses have confirmed improvement in local control but no improvement in OS. Recent studies using neo-adjuvant chemotherapy followed by concurrent chemoradiotherapy have produced encouraging early results, but longer term data are awaited.^19^^,^^20^

### The use of routine tumour markers in the management of NPC

Testing for Epstein–Barr virus (EBV) infection has potential as a screening tool, but only in high risk regions or populations. Epstein–Barr virus DNA testing has also been used as diagnostic and prognostic tool, and in the detection of recurrence. However, no consensus exists on either the appropriate cut-off values or the additional value to clinical management.

### Key points

• Nasopharyngeal carcinoma is frequent in patients of Southern China, Northern African and Alaskan origin, but in western countries the adjusted incidence is very low with only up to 1 per 100 000
• The most common signs and symptoms are nasal obstruction, epistaxis, conductive hearing loss secondary to otitis media with effusion, cranial nerve neuropathies and cervical lymphadenopathy
• Intensity modulated radiotherapy, with or without concurrent chemotherapy, is the mainstay of curative treatment, with concurrent chemotherapy for stage III and IV disease
• A positron emission tomography–computed tomography, computed tomography or magnetic resonance imaging scan should be carried out at three months from completion of treatment to assess response
• Salvage surgery should be considered for residual or recurrent disease at the primary site
• Controversy exists in the management of otitis media with effusion in patients with nasopharyngeal carcinoma, the best mode of salvage surgery, salvage treatments for disseminated disease, the use of neo-adjuvant and adjuvant chemoradiotherapy and the use of tumour markers.
